# SOLVe: a closed-loop system focused on protective mechanical ventilation

**DOI:** 10.1186/s12938-023-01111-0

**Published:** 2023-05-16

**Authors:** Philip von Platen, Philipp A. Pickerodt, Martin Russ, Mahdi Taher, Lea Hinken, Wolfgang Braun, Rainer Köbrich, Anake Pomprapa, Roland C. E. Francis, Steffen Leonhardt, Marian Walter

**Affiliations:** 1 Chair for Medical Information Technology, RWTH Aachen University, Aachen, Germany; 2 Department of Anesthesiology and Operative Intensive Care Medicine CCM CVK, Charité, Universitätsmedizin Berlin, Corporate Member of Freie Universität Berlin and Humboldt Universität zu Berlin, Berlin, Germany; 3 Fritz Stephan GmbH, Gackenbach, Germany; 4 EKU Elektronik GmbH, Leiningen, Germany; 5 Department of Anesthesiology, Friedrich-Alexander-Universität Erlangen-Nürnberg, Uniklinikum Erlangen, Erlangen, Germany

**Keywords:** Protective ventilation, Acute respiratory distress syndrome, Physiological closed-loop control

## Abstract

**Background:**

Mechanical ventilation is an essential component in the treatment of patients with acute respiratory distress syndrome. Prompt adaptation of the settings of a ventilator to the variable needs of patients is essential to ensure personalised and protective ventilation. Still, it is challenging and time-consuming for the therapist at the bedside. In addition, general implementation barriers hinder the timely incorporation of new evidence from clinical studies into routine clinical practice.

**Results:**

We present a system combing clinical evidence and expert knowledge within a physiological closed-loop control structure for mechanical ventilation. The system includes multiple controllers to support adequate gas exchange while adhering to multiple evidence-based components of lung protective ventilation. We performed a pilot study on three animals with an induced ARDS. The system achieved a time-in-target of over 75 % for all targets and avoided any critical phases of low oxygen saturation, despite provoked disturbances such as disconnections from the ventilator and positional changes of the subject.

**Conclusions:**

The presented system can provide personalised and lung-protective ventilation and reduce clinician workload in clinical practice.

## Background

Positive pressure mechanical ventilation remains the cornerstone of respiratory support and treatment for patients with acute respiratory distress syndrome (ARDS). Here, the mechanical ventilator supplies supplementary oxygen, allows respiratory rate and ventilation adjustment, and keeps the lung open by applying positive pressures (at inspiration and end-expiration). However, while mechanical ventilation is invaluable for keeping patients alive, it remains a double-edged sword. Namely, it bears the risk of damaging the lung further due to overdistention, cyclic alveolar opening and closing, and inflammation ensuing from mechanical stress and strain of lung tissue. Such injurious ventilation can lead to ventilator-induced lung injury (VILI) [1]. Hence, the application of lung-protective ventilator settings is crucial.

Choosing the correct ventilator settings for personalised and lung-protective ventilation requires experienced personnel with sufficient time to constantly observe and respond to a patient’s changing state. Yet, for many reasons, achieving this degree of therapy with continuous observation and instantaneous response for each patient is rarely possible [2, 3]. In addition, the number of experienced clinicians may be limited, especially in smaller, rural hospitals or during a pandemic like the recent Covid-19 pandemic. Computerised and closed-loop control systems are one possibility to mitigate these challenges [4].

The main therapeutic goals of mechanical ventilation are to provide adequate gas exchange while considering protective limits for pressure and volume. Adequate gas exchange includes oxygenation and alveolar ventilation, which are reflected by arterial partial pressures of oxygen (*P*aO$_2$) and carbon dioxide (*P*aCO$_2$), respectively. In addition, continuously available measurements, including peripheral oxygen saturation (*S*pO$_2$), end-tidal partial pressure of CO$_2$ (*P*ETCO$_2$), as surrogates of *P*aO$_2$ and *P*aCO$_2$, and pressure and flow measured at the airway opening (*p*$_\text {aw}$ and $\dot{V}_\text {aw}$), are being used to guide ventilator settings. Available ventilator settings include the fraction of inspired oxygen (*F*IO$_2$), the positive end-expiratory pressure (PEEP), respiratory rate ($f_\text {R}$), tidal volume (*V*$_\text {T}$) and the driving pressure ($\Delta P$). Tidal volume is often scaled to predicted body weight (pbw), resulting in *V*$_\text {T,pbw}$.

Several important clinical trials evaluated the correlation of different ventilator settings on the primary outcome measure of mortality rate (ARMA [5], ALVEOLI [6], LOVS [7], ExPress [8]) and secondary measures, such as ventilator-free days, organ failure-free days and oxygenation. Based on randomised controlled trials and extensive meta-analysis, strong evidence exists for using *V*$_\text {T,pbw}$ $\le 6\,\hbox {ml/kg}$ for ARDS patients. These recommendations have been included in local, such as the German *S3 guidelines on invasive ventilation and use of extracorporeal procedures in acute respiratory insufficiency* [9], and international guidelines, such as the Surviving Sepsis Campaign [10] or the ARDSNet protocol [5]. Clear evidence also exists for using a PEEP of at least 5 mbar and even higher PEEP [9–12]. However, no consensus on the optimal method to choose the PEEP exists [13, 14]. Amato et al. found the greatest correlation between the relative risk of death in the hospital and driving pressure ($\Delta P$) [15], leading to the recommendation of limiting $\Delta P$ $< 15\,\hbox {mbar}$ [9]. Application of the lowest necessary *F*IO$_2$ to keep patients within the *S*pO$_2$ targets is well-accepted. Oxygen toxicity and possible increased mortality with liberal oxygen targeting strategies have also been reported [16].

These recommendations and guidelines provide targets and upper limits for ventilator settings; however, continuous and personalised adherence is rarely possible in the clinical environment for several reasons. Computerised decision support systems (CDSS) are an option to improve adherence and personalise the ventilator settings by recommending ventilator settings to the clinician. The clinician must, however, be present to apply the change. Systems using heuristic rules [17], applying model-based optimisations using physiological models [18, 19], or a combination of both [20] have been presented. A CDSS can provide a deeper physiological representation of a patient’s state and improve the treatment strategy [18]. However, the requirement for a clinician’s presence remains a limitation in reducing the clinician’s workload.

A closed-loop system, which automatically, i.e., without needing a clinician’s presence, adjusts settings based on targets, feedback of measured values and a control algorithm, would be advantageous in this case. A CDSS or clinical protocol can be programmed to run in a closed-loop form. Examples would be the automatic ARDSNet protocol system [21] or the automation of the open lung concept [22]. Automatic closed-loop control are often focused on either oxygenation [23, 24] or ventilation (*P*ETCO$_2$) [25–27]. Highly automated systems which combine oxygen and carbon dioxide controllers have also been presented, see for example [28], or the commercially available system INTELLiVENT^®^-ASV (Hamilton Medical AG, Switzerland) [29]. We recently reviewed currently available physiological closed-loop control (PCLC) systems [4]. Lung-protective ventilation, as described above, typically played a secondary role for many of these systems compared to achieving the physiological setpoints. The clinical evidence is often more recent than the above-mentioned algorithms and, therefore, not included in their design.

In this paper, we present our **S**ystem for aut**o**matic **L**ung-protective **Ve**ntilation (SOLVe) with the aim to couple evidence-based protective guidelines with closed-loop control of mechanical ventilation. The system has defined protective operating ranges for ventilator settings, including adaptive limits, uses multiple closed-loop controllers and incorporates clinical knowledge into the controllers.

## Results

In this pilot study, the SOLVe system was evaluated in three animal subjects to demonstrate algorithmic performance. Table 1 shows the details of each experiment, during which the automated system ran for 6 h.

**Table 1:** Details of the *in-vivo* experiments

Subject	Respiratory failure model type	Number of disconnection	Tilting	Number of PEEP titrations	Compromise operating range allowed?
A	II	0	No	2	No
B	I	4	Yes	2	No
C	II	2	Yes	1	Yes

**Fig. 1:**
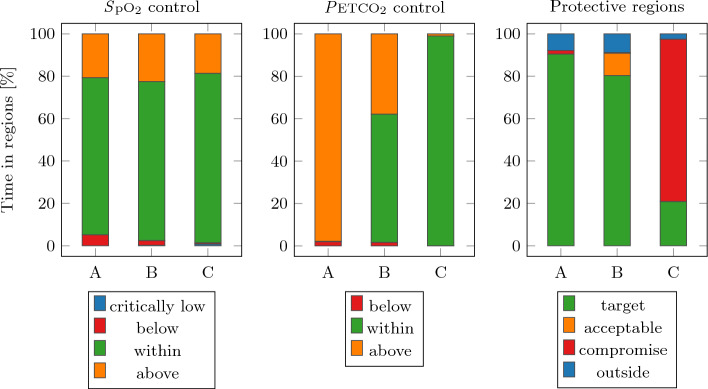
Percentage of time-in-target for all subjects

The percentage of time-in-target for each of the goals is shown in Fig. 1. The *S*pO$_2$ target was met for almost 80 % of the time in all three subjects. The *P*ETCO$_2$ was met for subject C for more than 95 % of the time, while subjects A and B spent considerably less time within the target region. Subjects A and B met the protective *target* region for over 80 % of the time, while subject C was in the protective *compromise* region for almost 80 % of the time.

Subject A had a $C_\text {rs,pbw}$ of $0.32 \pm 0.02 \, (\hbox {ml/kg})/\hbox {mbar}$ (mean ± standard deviation), which meant it was on the edge of the possible protective *target* region. This limitation on the minute ventilation made it impossible for the *P*ETCO$_2$ target to be met but validated the hierarchy defined in the controller structure that $\Delta P$ limitation trumps *P*ETCO$_2$. The $C_\text {rs,pbw}$ of subject B was the largest at $0.38 \pm 0.10 \, (\hbox {ml/kg})/\hbox {mbar}$, which allowed SOLVe to spend time in the *acceptable* target region and therefore achieve the *P*ETCO$_2$ target for more than 60 % while staying within the protective ranges for *V*$_\text {T,pbw}$ and $\Delta P$. For subject C, the $C_\text {rs,pbw}$ was $0.37 \pm 0.10 \, (\hbox {ml/kg})/\hbox {mbar}$, but the *compromise* target region was activated at the clinicians’ discretion. The *P*ETCO$_2$ target was met but with a more aggressive $\Delta P$ range.

**Fig. 2:**
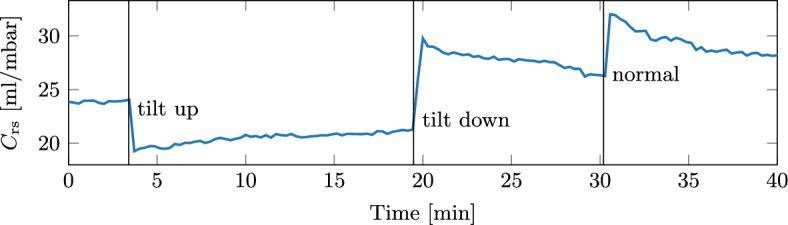
Tilting the subjects significantly changed their respiratory compliance. An example for subject B is shown here

The effect of tilting the subjects is shown for the exemplary case of subject B in Fig. 2. Tilting the subject up caused the $C_\text {rs}$ to decrease significantly. Tilting the subject’s head down resulted in a large increase in $C_\text {rs}$  and subsequently returning to the normal position increased $C_\text {rs}$ even further.

### Automated PEEP titration

An automated PEEP titration was initiated at the beginning of each experiment. For subjects A and B, a second PEEP titration was initiated by the clinician after 4 h of closed-loop mechanical ventilation. In all cases, the *best* PEEP was identified automatically, and no clinician interaction was required. The change in compliance between before and after the automated PEEP titration is given in Table 2. After the titration, the identified *best* PEEP was higher for two trials, lower for two trials and the same for one trial compared to before the titration. In all cases, the compliance was the same or higher after the PEEP titration.

**Table 2:** PEEP and compliance values before and after the automated PEEP titration

Subject	Trial	Before titration		After PEEP titration		Difference	
		PEEP	$C_\text {rs}$	PEEP	$C_\text {rs}$	$\Delta$PEEP	$\Delta$ $C_\text {rs}$
		[mbar]	[ml/mbar]	[mbar]	[ml/mbar]	[mbar]	[%]
A	1	15	15	16	15	+1	0.0
A	2	18	13	14	16	– 4	23.1
B	1	12	13	14	16	+2	23.1
B	2	14	17	14	24	0	41.2
C	1	15	17	14	18	– 1	5.9

### *S*pO$_2$ control results

**Fig. 3:**
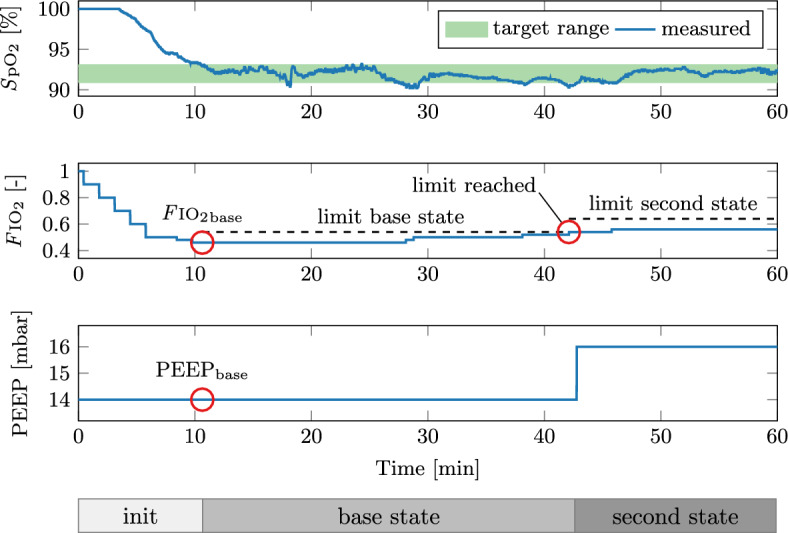
Dynamic response of the *S*pO$_2$ controller for subject A. The bars at the bottom show the current *S*pO$_2$ controller state

In addition to the time-in-target results, the dynamic response of the *S*pO$_2$ was evaluated. As an illustrative example, Fig. 3 shows a section where *S*pO$_2$ controller was particularly active in subject A. After the initial phase, where the *F*IO$_2$ was titrated down from 1.0, the *base* values were found. Small *F*IO$_2$ increases occurred continuously to keep the *S*pO$_2$ within the target range. At $t = 42\,\hbox {min}$ the *F*IO$_2$ reached the *base* limit and the controller moved to the *second* region. This also included a PEEP increase, which stabilised the *S*pO$_2$ within the target region again. The new *F*IO$_2$ limit is also shown.

**Fig. 4:**
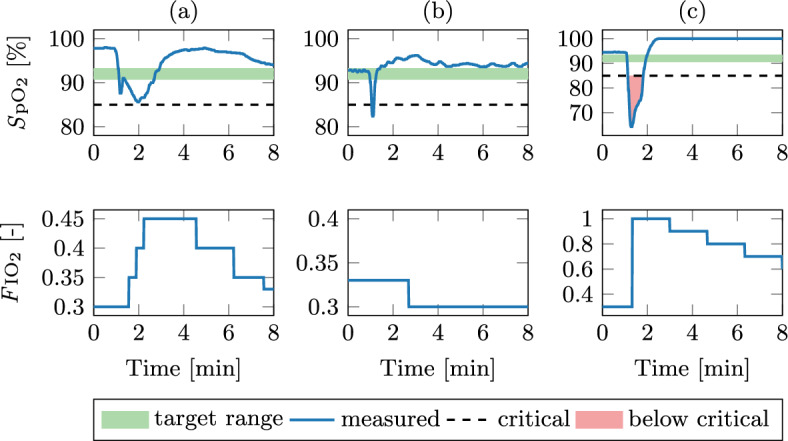
Dynamic response of the *S*pO$_2$ controller after disconnection from the mechanical ventilator

Brief disconnections (15 s) between the subject and the mechanical ventilator were performed and three such cases are shown in Fig. 4.

In each case, the disconnection causes a significant decrease in *S*pO$_2$. For case (a), the lowest *S*pO$_2$ value was 85.6 % and the *S*pO$_2$ was below the target for 100 s. In case (b), no intervention of the *S*pO$_2$ controller was needed because the *S*pO$_2$ recovered in 10 s after reconnection. The *S*pO$_2$ subsequently overshot the target, to which the *S*pO$_2$ controller responded by decreasing the *F*IO$_2$. Case (c) represents the worst-case, as the *S*pO$_2$ drops below the critical value of 85 %. Here, the controller correctly responded by setting the *F*IO$_2$ to 1.0. The lowest *S*pO$_2$ value was 63.4 %, the *S*pO$_2$ was below the critical value for 30 s and below the target for 40 s.

### *P*ETCO$_2$ control and PRVC results

**Fig. 5:**
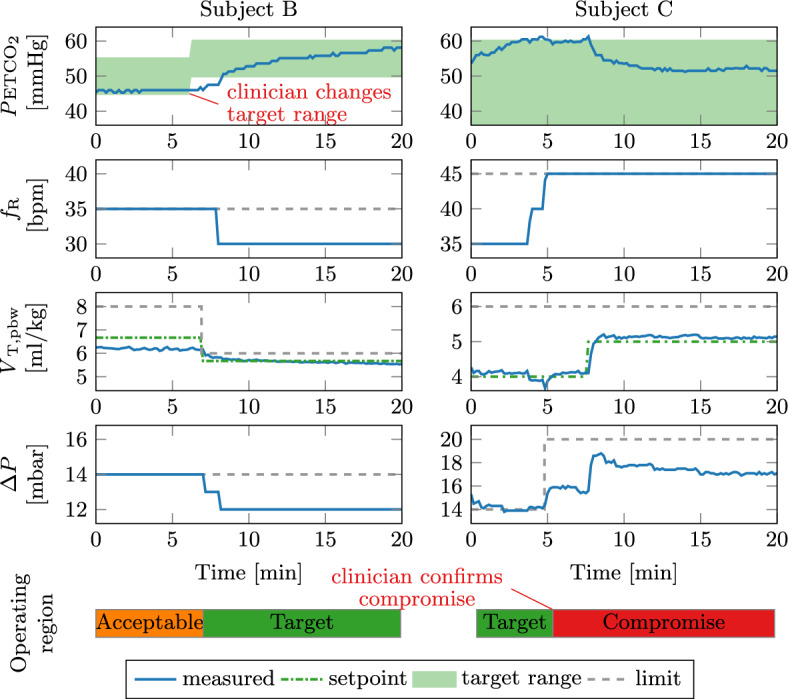
Dynamic response of the *P*ETCO$_2$ and PRVC controllers for subjects B (left) and C (right). The bars at the bottom show the current operating region of the controller. The red text shows clinician interaction. The green dot-dashed line shows the *V*$_\text {T,pbw,target}$ (setpoint) for the inner control loop. The grey dashed lines define the limits as given by the operating region

For illustration, the dynamic responses of the cascaded *P*ETCO$_2$ and PRVC control are shown for selected situations in Fig. 5.

The clinician adapted the *P*ETCO$_2$ target range at $t = 6\,\hbox {min}$ for subject B. Since the *P*ETCO$_2$ was below the new target range and the current *acceptable* operating range was active, SOLVe automatically decreased the* V*$_\text {T,pbw,target}$ first. The PRVC controller adapted $\Delta P$ such that the new *V*$_\text {T,pbw,target}$ was achieved, thereby also moving into the protective *target* region. The $f_\text {R}$ was also automatically decreased since the *P*ETCO$_2$ was still below the target even after the *V*$_\text {T,pbw,target}$ change. This brought the *P*ETCO$_2$ into the target range.

For subject C, the *P*ETCO$_2$ exceeded the target range after $t = 3\,\hbox {min}$, to which the controller responded by increasing the $f_\text {R}$ rate twice. Subsequently, the *V*$_\text {T,pbw}$ dropped below 4 ml/kg  which alerted the clinician to consider activating the *compromise* region. After the confirmation, the PRVC controller increased the $\Delta P$ to ensure *V*$_\text {T,pbw}$= 4 ml/kg. At $t = 7\,\hbox {min}$, the *P*ETCO$_2$ again left the target range, but the $f_\text {R}$ rate was already at the maximum. Therefore, the *P*ETCO$_2$ controller increased the *V*$_\text {T,pbw,target}$ to 5 ml/kg.

## Discussion

We designed a closed-loop control expert system that automatically adapts all ventilator settings to achieve the *S*pO$_2$, *P*ETCO$_2$, and lung protective targets recommended for mechanical ventilation in ARDS patients. A pilot study for the SOLVe system showcases its accuracy, robustness and reliability. The system achieved especially good time-in-target results for the *S*pO$_2$ control and avoided any critical phases of de-saturation despite provoked disturbances. The recommended protective limits were adhered to at all times, and, if possible, the *P*ETCO$_2$ was also regulated. As such, the system can reliably help the clinician keep mechanical ventilation safe and could drastically reduce the workload of clinical staff responsible for observing and adjusting the mechanical ventilator settings. In each case, the ventilator settings were also highly personalised and continuously adapted to respond to variable disease states and conditions and to the various artificial disturbances.

### Automated PEEP titrations

Decremental PEEP titrations finding the maximum compliance, as applied by the SOLVe system, are only one of many methods proposed for optimising PEEP. However, no consensus on the optimal method currently exists [13, 14]. An early landmark study by Suter et al. proposed titrating PEEP so that oxygen delivery is maximised [30]. The calculation of oxygen delivery, however, requires invasive measurement of cardiac output and oxygen tension. Respiratory system compliance was proposed as a substitute, since it correlated well with oxygen delivery. Suarez-Sipmann et al. showed that compliance could be used to identify the beginning of collapse after recruitment and confirmed these findings with oxygenation and computed tomography scans [31]. Therefore, the chosen approach for the SOLVe system aligns with the current clinical practice. However, unlike current manual PEEP titrations, which often go way past the *best* PEEP to identify it, the SOLVe system stops automatically at the *best* PEEP. Therefore, no additional recruitment manoeuvres are necessary after the PEEP titration, as was done in a large clinical trial [32]. As a secondary benefit, automatically titrating PEEP to maximise $C_\text {rs}$ enlarges the operating region for *V*$_\text {T,pbw,target}$ and $\Delta P$ and remains invaluable for the subsequent protective control.

### *S*pO$_2$ control

The *S*pO$_2$ controller was shown to be dynamic, safe and highly personalised and achieved a time-in-target of over 80 % and avoided critical de-saturation events.

Recent publications [16, 33] highlight the importance of oxygen control and avoiding both hyperoxemia and hypoxemia. Excessive oxygen over long periods can be toxic, and links between liberal *F*IO$_2$ and increased mortality have been presented [16]. Hypoxemia can lead to irreversible tissue damage, neurological damage or death and must be avoided at all costs [33].

Achieving a tight oxygen control requires many adaptions of *F*IO$_2$, as shown in Fig. 3, which would place a high burden on clinical staff. The presented *S*pO$_2$ controller performed this task reliably and robustly, even during disturbances. Different sample times for phases above and below the target and small changes of *F*IO$_2$ in steps of 0.02 allowed for fast and dynamic yet robust feedback control of *S*pO$_2$. The small *F*IO$_2$ changes differ from the ARDSNet table recommendation of steps of 0.1. These large *F*IO$_2$ changes could lead to oscillatory behaviour around the target—or having to accept remaining above the target with excessive *F*IO$_2$.

The approach to defining controller states for coupling the *F*IO$_2$ and PEEP incorporates important clinical knowledge into the controller. Previous computerised systems have also included a coupling between these variables, such as evaluating the PEEP/*F*IO$_2$ ratio to determine if PEEP changes should occur [34] or using the ARDSNet protocol as guidance (INTELLiVENT^®^-ASV) [29]. Importantly, however, the SOLVe system bases the level of PEEP first on optimising compliance and second on oxygenation. Furthermore, the pairing of *F*IO$_2$ and PEEP is personalised by automatically finding a suitable base pairing of *F*IO$_2$ and PEEP. No limits exist for this initial base pairing, meaning it is individualised to fit the diverse patient and disease populations in the clinical environment. All further pairings are then based on this initial base pairing.

### *P*ETCO$_2$ and PRVC control

The default target for the *P*ETCO$_2$ was chosen to be 30 mmHg to 60 mmHg, which is within the limits given in *German S3 guidelines* for permissive hypercapnia [9]. This target was only partially achievable for subjects A and B without infringing on the lung-protective limits of $\Delta P$. Once the target for *P*ETCO$_2$ could not be met without infringing the $\Delta P$ limit, the clinician was alerted of the conflict. For subjects A and B, the clinician decided not to increase the PRVC controller’s operating region leading to *P*ETCO$_2$ greater than 60 mmHg and hypercapnia outside the target range. This was tolerated in the animal experiments at the discretion of the attending clinician, and there was no hard cut-off for *P*ETCO$_2$ in this pilot study. Instead, the goal was to test whether the higher-priority lung-protective ventilator settings were adhered to. This limitation within the algorithm was a core consideration during the design of the rule-based control law. In a comparable clinical situation, the clinical team would evaluate other therapeutic options, such as prone positioning, recruitment manoeuvres, or extra-corporeal CO$_2$ removal, before increasing the aggressiveness/invasiveness of mechanical ventilation.

In any case, the SOLVe system always ensures that the applied $\Delta P$ is within the protective limits and only goes beyond this limit after clinical confirmation—thus allowing the clinician to consider other therapeutic options first. Through visual messages, the clinician is aware of this conflict and can make better decisions. Hence, the SOLVe system can be used as an automated tool to assist the clinician in providing protective ventilation and reaching the therapeutic targets without the automation interfering with the clinical decisions.

The *P*ETCO$_2$ control in the SOLVe system is based on simple rules and clinical expertise. Nonetheless, the controller held the *P*ETCO$_2$ within the target most of the time without infringing on protective limits. Importantly, this highlights the hierarchy that $\Delta P$ is the most critical parameter, and the simplicity of the rules makes the controller more transparent and understandable for clinical staff.

We note that *P*ETCO$_2$ is used as a surrogate for *P*aCO$_2$  which would be the ideal measurement for CO$_2$ control. However, *P*aCO$_2$ is only available with discrete invasive blood gas analysis. While the difference between *P*ETCO$_2$ and *P*aCO$_2$ is non-linear and time-varying, studies have shown that *P*ETCO$_2$ and *P*aCO$_2$ correlate to a certain degree [35]. Our application used every available *P*aCO$_2$ from an hourly arterial blood gas analysis to confirm that the current *P*ETCO$_2$ target range was acceptable. Changes to the *P*ETCO$_2$ target could have been made if the discrepancy became too large. Adjusting alveolar ventilation to the arterial pH, as recommended by the ARDSNet protocol, was deemed impractical due to the discontinuity of the parameter and the lack of an adequate surrogate.

The upper limit of 45 breaths per minute for the respiration rate set by the clinicians at the beginning of all experiments is above the common 35 breaths per minute [5]. This value was allowed to be higher due to the experiments being performed on adolescent pigs with a healthy resting breathing rate of 37±12 breaths per minute [36]. This limit would be set for future clinical applications to a value appropriate for the patient. Due to the stiff respiratory mechanics (small $C_\text {rs}$), the time constant of the respiratory system ($\tau$) was never greater than 0.22 s, and the upper limit for $f_{max}$ remained at 45 for all experiments. All subjects had complete expiration, and no build-up of intrinsic PEEP was observed.

An active research area is the mechanical power and respiratory work applied during mechanical ventilation. A system by Becher et al. automatically determined the respiratory rate and tidal volume to minimise mechanical power [37]. However, the clinician had to choose the required minute ventilation in their system. The ASV mode (Hamilton Medical AG, Switzerland) and the FLEX system [20] continuously update the breathing rate to minimize the respiratory work to expedite weaning. Van der Staay et al. have recently provided a review of these concepts [38]. In the future, the PRVC and *P*ETCO$_2$ controllers of the SOLVe system could be extended to optimise for minimum mechanical power.

### Limitations

The experimental validation has the following limitations. Firstly, the system was only tested in a pilot study of three animal subjects. An experimental ARDS was induced in the animals, mimicking ARDS’s lung mechanical and functional characteristics [39]. Nonetheless, respiratory system compliance was significantly lower than reported in most literature on ARDS patients. This mostly led to the subjects being ventilated with very low tidal volumes (4 ml/kg) and $\Delta P$ in the range of 13 mbar to 20 mbar. Additionally, the respiratory frequency was also in the upper region of $40 \pm 5\hbox { bpm}$ to keep the subjects within the *P*ETCO$_2$ target for as long as possible. All animals required low to moderate *F*IO$_2$ levels to correct the hypoxaemia. Despite these challenges, the SOLVe system was still able to ventilate the subjects protectively and safely. The system is expected to act equally well in subjects with larger respiratory system compliance and more severe hypoxaemia.

## Conclusion

The SOLVe system is a closed-loop control system which automatically adapts all relevant ventilator settings to achieve the *S*pO$_2$, *P*ETCO$_2$, and protective targets recommended for mechanical ventilation in ARDS patients. An automatic PEEP titration algorithm finds the *best* PEEP and optimises the operating region for the subsequent protective ventilation. The system was evaluated in vivo and was shown to be safe and reliable. It is predicted that the system would (1) lead to a better application of the current clinical evidence for mechanical ventilation, (2) provide optimal adherence to current guidelines on protective ventilation, (3) provide a personalised therapy that is continuously adapted to the patient’s needs and (4) reduce clinicians’ workload to implement their therapeutic strategy for each patient.

## Materials and methods

**Fig. 6:**
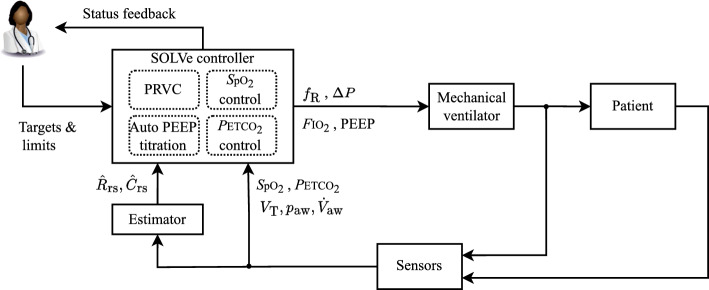
Block diagram of the SOLVe algorithm. PRVC stands for pressure-regulated volume control

SOLVe addresses the two main targets of mechanical ventilation strategies: gas exchange and protective ventilation. Since this is a multiple-input and multiple-output problem, four controllers and various continuous physiological measurements are included in the SOLVe system. An estimator provides online parameter estimates for a respiratory mechanics model (resistance $\hat{R}_\text {rs}$ and compliance $\hat{C}_\text {rs}$). A block diagram representation of the complete system is given in Fig. 6, showing the controllers, sensors and actuator, and the bi-directional user interface with the clinician. Table 3 shows the pairing of target, measurement and actuation.

**Table 3:** Targets, sensors and actuating variables in mechanical ventilation

Target	Measurement	Controller	Actuation
Oxygenation	*S*pO$_2$	*S*pO$_2$ controller	*F*IO$_2$, PEEP
Ventilation	*P*ETCO$_2$	*P*ETCO$_2$ controller	*V*$_\text {T,pbw}$, $f_\text {R}$
Protective volume and pressure	*V*$_\text {T,pbw}$, *p*$_\text {aw}$, PEEP	Pressure-regulated volume control	$\Delta P$, *V*$_\text {T,pbw}$
Best PEEP	$\dot{V}_\text {aw}$, *p*$_\text {aw}$, $\hat{C}_\text {rs}$	Automatic PEEP titration	PEEP

**Fig. 7:**
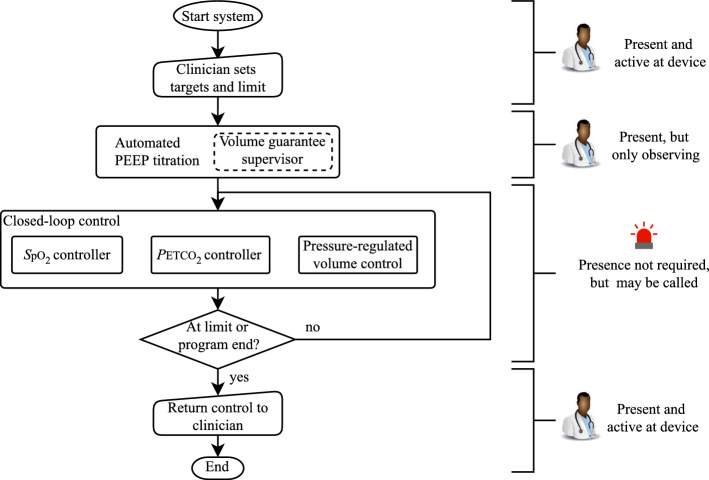
Flowchart showing the phases of SOLVe, as well as the active controllers and the clinician’s activity

The phases of operation of the SOLVe system and the interaction with the clinician are given in Fig. 7. Importantly, interaction with the clinician remains vital for the SOLVe system, even if the goal is to reduce this to a minimum. The clinician initially inputs targets and limits and observes the PEEP titration. During the closed-loop control, the clinician does not have to be present. Three controllers run in parallel at different update frequencies during this phase, as described later. The SOLVe system has a set of degrees and limits of freedom while keeping ventilation protective; if the system cannot achieve adequate gas exchange and ventilation within protective limits, the system alerts the clinician. They may be able to increase the operating space of SOLVe, or SOLVe may have reached a limit, at which point the clinician takes over control.

### System setup

Our prototype system consists of a real-time PC (MicroLabBox, dSPACE GmbH, Paderborn, Germany) connected to a medical panel PC ($\hbox {THA.leia}^3$, MCD Medical Computers Deutschland GmbH, Mönchengladbach, Germany) running MATLAB 2017b (The MathWorks Inc., Natick, USA) and dSPACE Control Desk version 7.1 (dSPACE GmbH, Paderborn, Germany). A modified mechanical ventilator (EVE, Fritz Stephan GmbH, Gackenbach, Germany) receives remote commands and sends all measurement data via a custom RS232 protocol to the real-time PC. The ventilator features a built-in pulse oximeter (MASIMO Rainbow, Irvine, USA) and a proximal mainstream capnograph (MASIMO IRMA CO2, Irvine, USA). All ventilation data (pressure, flow, and ventilation phase) are sampled at 100,Hz, while the other variables, such as *S*pO$_2$, pulse rate, and *P*ETCO$_2$ are sampled at 1 Hz. All data processing and control algorithms are executed on the real-time PC at a sampling rate of 100 Hz.

### Defining protective operating ranges

Achieving both protective ventilator settings and sufficient gas exchange may not be possible for all critically ill patients with ARDS. Therefore, operating ranges defining the limits and permitted degrees of freedom for the ventilator settings are mandatory and have been included in the SOLVe system.

SOLVe cannot replace the standard multimodal therapy approach for patients with ARDS, which, e.g., includes antibiotic treatment and fluid management. Other supplementary therapeutic options beyond ventilation, such as prone positioning or extra-corporeal membrane oxygenation, should always be considered according to the patient’s status, especially if a patient does not fall within the SOLVe operating range.

**Fig. 8:**
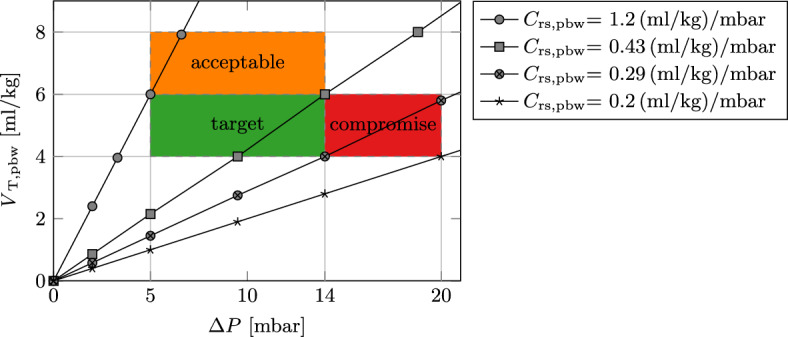
Protective operating regions for the SOLVe system

A first operating range is based on *V*$_\text {T,pbw}$ and $\Delta P$ and divided into three regions: *target*, *acceptable* and *compromise* as shown in Fig. 8.

The *target* region spans the area defined by clinical recommendations for tidal volume and driving pressure, *V*$_\text {T,pbw}$ $\le 6\,\hbox {ml/kg}$ and $\Delta P$  $\le 14\,\hbox {mbar}$ [9, 10], respectively. A lower limit of *V*$_\text {T,pbw}$ $\ge 4\,\hbox {ml/kg}$ ensures minimum alveolar ventilation of the patient, and $\Delta P$ $\ge 5\,\hbox {mbar}$ is a ventilator device limitation. To prevent consequences of severe hypercapnia (excessive CO$_2$), the *acceptable* region allows for larger yet safe tidal volumes of up to 8 ml/kg and still strictly limits the $\Delta P$. The *compromise* region allows $\Delta P$ $\le 20\,\hbox {mbar}$ to facilitate minimum alveolar ventilation even for patients with very stiff respiratory mechanics. However, unlike the *target* and *acceptable* region, explicit activation is required by the attending clinician since it falls outside of clinical recommendations [9, 15].

SOLVe applies a fast method to determine the probable operating point for each patient and situation by considering the respiratory mechanics. The passive respiratory system (without spontaneous breathing) can be modelled as a linear first-order system given by the equation of motion:

1 $$\begin{aligned} p_\text {aw}(t) = R_\text {rs} \cdot \dot{V}_\text {aw}(t) + \frac{1}{C_\text {rs}} \cdot V_\text {aw}(t) + p_\text {0} \quad , \end{aligned}$$

where $p_\text {aw}(t)$ is the airway pressure, $\dot{V}_\text {aw}(t)$ is the airway flow, $V_\text {aw}(t)$ is the volume, $R_\text {rs}$ is the respiratory resistance, $C_\text {rs}$ the respiratory compliance and $p_\text {0}$ is the total pressure at end expiration.

$\Delta P$ and *V*$_\text {T}$ are proportionally linked by respiratory compliance since $V_\text {T} \approx C_\text {rs} \cdot \Delta P$, if airway flow returns to zero at the end of inspiration. Since tidal volume is normally scaled to the patient’s predicted body weight, compliance can also be scaled to the predicted body weight ($C_\text {rs,pbw}$). This linear relationship is also plotted in Fig. 8 and shows the minimum or maximum $C_\text {rs,pbw}$ span for the operating regions outlined above. Patients with a $C_\text {rs,pbw}$ $< {0.2}\,(\hbox {ml/kg})/\hbox {mbar}$ cannot be ventilated within the defined regions and do not qualify for application of the SOLVe system, and other therapeutic options should be considered.

The operating range for the respiratory rate is defined to prevent a build-up of intrinsic PEEP and possible barotrauma. Initially, the clinician defines an upper and lower limit for $f_\text {R}$. Thereafter, the SOLVe system observes the time constant of the respiratory system ($\tau = R_\text {rs} \cdot C_\text {rs}$) and reduces the upper $f_\text {R}$ limit ($f_\text {max}$) to prevent intrinsic PEEP. Assuming an exponential pressure decay during the passive expiration, the computed $\tau$ is used to estimate the required expiration time. An exponential decay is almost complete after a time of  $T = 3\cdot \tau$, meaning complete expiration has occurred and the expiration flow is very close to zero. Keeping the inspiration to expiration ratio constant at 1:1, as recommended by [9], expiration must be complete within half a breath period. Therefore, an upper limit for $f_\text {R}$ can be calculated by:

2 $$\begin{aligned} f_\text {max} = \frac{60}{2\cdot \left( 3\cdot \tau \right) } \, . \end{aligned}$$

Furthermore, automatic checks for the build-up of intrinsic PEEP use are included. First, intrinsic PEEP is identified using the online estimation of the $\hat{p}_\text {0}$ value (see eq. 1) according to the method by Nucci et al. [40]. Second, complete expiration was observed by ensuring the expiratory flow was zero before the next breath started. If either method detects an intrinsic PEEP, the system automatically reduces the $f_\text {max}$ by 5 bpm and alerts the clinician.

The respiratory system parameters ($\hat{R}_\text {rs}$ and $\hat{C}_\text {rs}$) and total pressure at end expiration ($\hat{p}_\text {0}$) are calculated on a breath-by-breath basis using the equation of motion (eq. 1) by means of least squares estimation [41]. The linear first-order model was robust and sufficiently accurate for our application.

### Automated PEEP titration

**Fig. 9:**
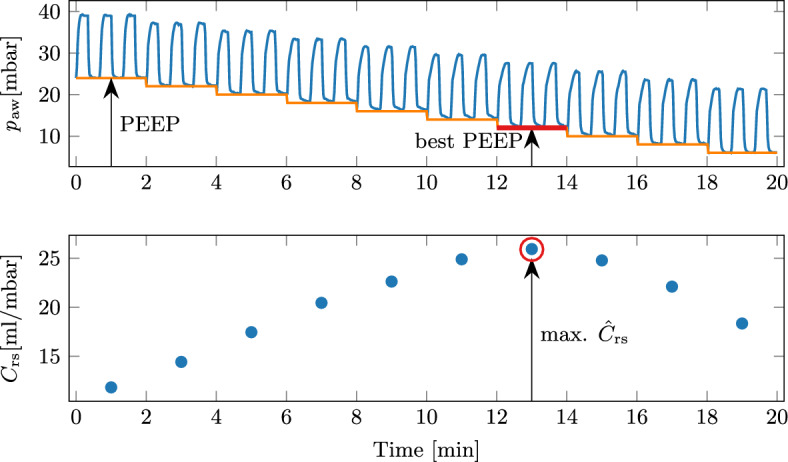
Exemplary PEEP titration to find the PEEP value with the maximum compliance (defined as the *best* PEEP). Three breaths on each level are indicative only, as the actual number of breaths depends on the $f_\text {R}$

Different methods of finding an adequate PEEP level have been proposed without consensus on the optimal method [13]. One such method is finding the PEEP at which the $C_\text {rs}$ is greatest [13]. Setting adequate PEEP to avoid derecruitment can significantly improve and maintain the compliance of the lungs. The procedure for finding the maximum compliance involves first increasing the PEEP level to a certain value (e.g., 24 mbar) and then step-wise reducing (titrating) the PEEP and observing the compliance at every level. This procedure is graphically shown in Fig. 9. Importantly, decremental instead of incremental titrations should be used due to the hysteresis of the lungs [42].

This procedure was automated in the SOLVe system. To start the procedure and avoid a single large step, the system automatically increases the PEEP in steps of 4 mbar until the starting pressure 24 mbar is reached. Each PEEP level is held for 2 min, while the median $\hat{C}_\text {rs}$ is calculated and evaluated. A logistic regression classifier evaluates whether the current PEEP level is the *best*. It has previously been presented in detail [43]. Briefly, the relative change in $\hat{C}_\text {rs}$ compared to the previous PEEP level is calculated and classified into one of three classes:

3 $$\begin{aligned} q(PEEP) = {\left\{ \begin{array}{ll} 1, &{} {best}\, \text {PEEP } \\ 0 ,&{} \text {above }{best} \, \text {PEEP } \\ -1 ,&{} \text {below }{best}\, \text {PEEP } \end{array}\right. } \end{aligned}$$

If the current PEEP level is classified as **above** ***best*** **PEEP**, the PEEP is reduced by 2 mbar and is re-evaluated. This procedure is repeated until either ***best*** PEEP or **below** ***best*** **PEEP** are found and the titration procedure ends. If ***best*** PEEP is reached, the relative compliance change is small enough, meaning the peak of the PEEP versus $\hat{C}_\text {rs}$ curve has probably been reached. Reaching the **below** ***best*** **PEEP** means the compliance has decreased and the *best* PEEP was on the previous level. PEEP is increased to the previous level before the titration ends.

The titration is performed in a pressure-controlled mode with a $\Delta P$ of 14 mbar. A volume guarantee supervisor ensures that the *V*$_\text {T,pbw}$ remains between 4 ml/kg and 8 ml/kg, and can vary the $\Delta P$ if needed. Finally, a lower limit of 6 mbar for the PEEP exists, as recommended in [9]. To prevent hypoxemia, *F*IO$_2$ is kept at 1.0 during the PEEP titration. In the case of a rapidly falling *S*pO$_2$, a safety routine is initiated to stabilise the patient.

### *S*pO$_2$ controller

The *S*pO$_2$ controller utilises both *F*IO$_2$ adjustments and PEEP increases to achieve the *S*pO$_2$ target, which the clinician provides as a range between *target high* and *target low*.

**Fig. 10:**
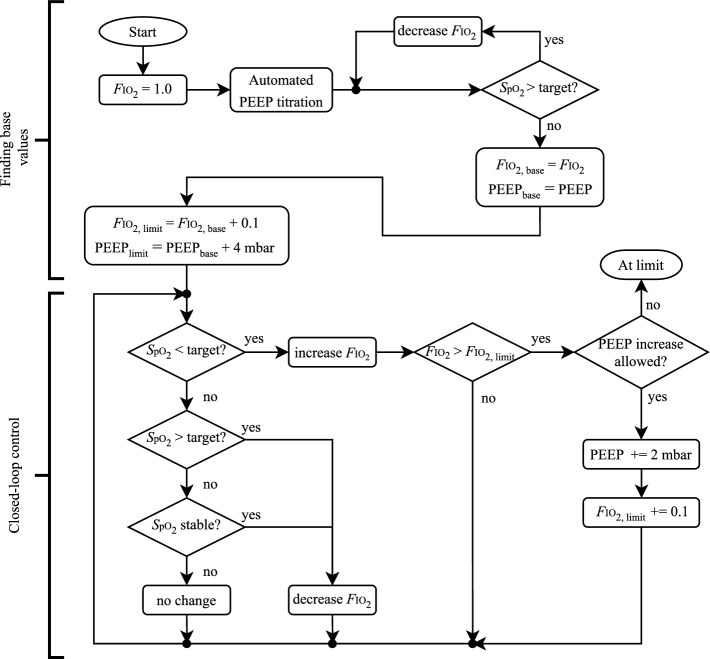
Rules for the *S*pO$_2$ controller

Before starting the closed-loop control, an automatic initialisation phase finds a base pairing of PEEP and *F*IO$_2$. The PEEP$_\text {base}$ is found using the automated PEEP titration to maximise compliance. Following this, the *F*IO$_2$ is decreased from 1.0 until the *S*pO$_2$ target is reached, as shown in Fig. 10.

Subsequently, automatic adjustments in *F*IO$_2$ depend on measured *S*pO$_2$ as shown in Fig. 10 and Table 4. Small *F*IO$_2$ adjustments ensure the target can be reached without excessive overshoot. While above the target, the reduced update rate (100 s) prevents limit cycles or undershooting the target. Below the target, a faster response is required; hence a faster update rate of 20 s is used. If the *S*pO$_2$ falls below the lower limit (85%), the *F*IO$_2$ is immediately set to 1.0 to prevent hypoxemia. Once a patient has remained stable within the upper target range for a predefined period (20 min for our case), with no increases in *F*IO$_2$ or PEEP, the expert system slowly decreases the *F*IO$_2$ until the patient is at the lower end of the target. Thereby, the *F*IO$_2$ is reduced to a minimum, preventing risks of an unnecessarily high *F*IO$_2$.

**Table 4:** Update rules for *F*IO$_2$ based on current *S*pO$_2$ value

*S*pO$_2$	Description	Update rule	Update time
> high target + 4	Greatly above target	Decrease *F*IO$_2$ by 0.1	100 s
> high target + 2	Above target	Decrease *F*IO$_2$ by 0.05	100 s
> high target	Slightly above target	Decrease *F*IO$_2$ by 0.02	100 s
> target middle	Upper target region	If stable: decr. 0.02, else: no change	20 min
> low target	Lower target region	No change	–
< low target	Slightly below target	Increase *F*IO$_2$ by 0.02	40 s
< low target -2	Below target	Increase *F*IO$_2$ by 0.05	20 s
$\le$ 85	*S*pO$_2$ low state	Set *F*IO$_2$ to 1.0	Immediately

In addition to increasing *F*IO$_2$, increasing PEEP is a valid therapeutic measure if the *S*pO$_2$ becomes low. A higher PEEP increases the lung’s functional residual capacity and can prevent alveolar derecruitment, reduce atelectasis and improve gas exchange.

Therefore, if a significant decline in the gas exchange capability of the lungs has occurred since the base pairing of PEEP and *F*IO$_2$ was found, the current PEEP may be insufficient. To avoid conflicts between the *best* PEEP based on maximum compliance (automated PEEP titration above) and increasing PEEP based on oxygenation, a set of conditions for PEEP increases were designed:

- No increase in PEEP for the first 60 min after a PEEP titration manoeuvre.
- PEEP can only increase in steps of 2 mbar.
- Only two increases are allowed per 24 h interval.
- There should be 5 min between subsequent PEEP increases to allow for the change to have an effect [44].

The coupling and the evaluation of whether a PEEP increase should occur are also included in the flowchart in Fig. 10. If the above conditions are not satisfied, the SOLVe algorithm has reached a limit and issues an alarm. Clinical staff must evaluate the patient’s condition at the bedside and take appropriate therapeutic actions.

For better visualisation to the user, the three operating states of the controller are given names as shown in Table 5. The automatic change between states is sequential, i.e. base $\rightarrow$ second $\rightarrow$ final, and non-reversible. The system does not automatically move down to a lower state because PEEP reductions bear the risk of alveolar derecruitment and deterioration of gas exchange. Therefore, a decision to reduce PEEP requires the clinician’s attention.

**Table 5:** Definitions of the *S*pO$_2$ controller states

State	Upper *F*IO$_2$ limit	PEEP
Base	*F*IO$_{2\text {,base}}$ $+ \, 0.1$	PEEP$_\text {base}$
Second	*F*IO$_{2\text {,base}}$ $+ \, 0.2$	PEEP$_\text {base} + 2\,\hbox {mbar}$
Final	*F*IO$_{2\text {,base}}$ $+ \, 0.3$	PEEP$_\text {base} + 4\,\hbox {mbar}$

Movement between states is sequential and non-reversible

### Pressure-regulated volume control and *P*ETCO$_2$ control

**Fig. 11:**
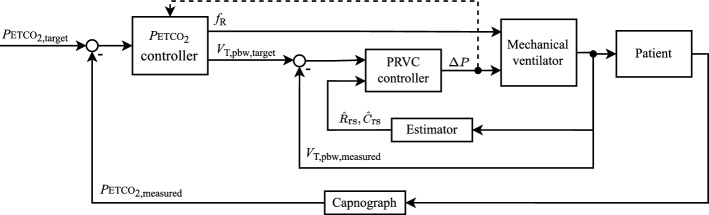
Block diagram of the cascaded control loop for the control of *P*ETCO$_2$ and *V*$_\text {T,pbw}$

A cascaded control loop is used for the control of *P*ETCO$_2$ and *V*$_\text {T,pbw,target}$ as shown in Fig. 11. The clinician inputs a *target high* and *target low* to define a *P*ETCO$_2$ target range. Note that the *V*$_\text {T,pbw,target}$ is calculated automatically by the system and cannot be adapted by the clinician.

The inner loop contains the pressure-regulated volume control, which adjusts $\Delta P$ to achieve the V$_\text {T,pbw,target}$. Here, a proportional-integral (PI) controller with feed-forward is used. The inverse of the estimated respiratory system model is used for the feed-forward element, while the PI controller eliminates any steady-state error. The $\Delta P$ is updated on a breath-by-breath basis and limited to a maximum change of 3 mbar per breath.

**Fig. 12:**
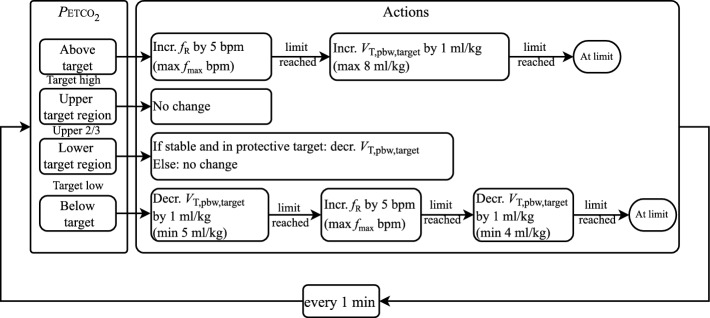
Update rules for the *P*ETCO$_2$ controller. *V*$_\text {T,pbw,target}$ is only updated if the current $\Delta P$ value allows this—see the section on defining a protective operating range

The outer loop regulates the *P*ETCO$_2$ by adapting the $f_\text {R}$ and *V*$_\text {T,pbw,target}$. The actual controller is rule-based and contains the rules as shown in Fig. 12. This outer loop runs with a sampling time of 1 min. The upper limit for the respiration rate ($f_\text {max}$) is defined based on the operating range explained in the earlier section.

### In vivo experiments

A pilot study used the SOLVe system to ventilate adolescent pigs with respiratory failure (German landrace, male, approx. 40 kg). The study was approved by the Institutional Animal Care and Use Committee (Tierversuchskommission, Landesamt für Gesundheit und Soziales, Berlin, Germany; approval number G 0229/18), and all animal procedures were conducted complying with national regulations and institutional animal care committee guidelines.

All animals were placed in the horizontal supine position at the beginning of the experiment. They were put under general anaesthesia and instrumented before a baseline phase was started. Two different models for respiratory failure induction were used [39]. For model I, surfactant depletion by saline-based lung lavages was used. For model II the combination of surfactant depletion and injurious ventilation was used. The Berlin definition of severe ARDS, i.e. a *P*aO$_2$/*F*IO$_2$ $\le$ 100 mmHg with PEEP $\ge$  5 cmH$_2$O, was used to quantify the respiratory failure before starting the SOLVe system. First, the clinical targets and limits of ventilation were set according to clinical reasoning. Then, 6 h of automated ventilation using SOLVe were started. The animals remained deeply sedated throughout the experiment and had no spontaneous breathing.

The goal for the system was to remain within target ranges for *S*pO$_2$, *P*ETCO$_2$ and protective ventilation for as long as possible without any interaction from the clinician. The default targets for the system are given in Table 6. The *S*pO$_2$ target was also considered met if the *S*pO$_2$ was above the target, but the *F*IO$_2$ was at the lower limit of 0.3. Furthermore, it was considered critical if the *S*pO$_2$ dropped below 85 %. The targets were adapted during the experiments to test the controllers.

**Table 6:** Default targets for the SOLVe algorithm as used during the in vivo experiments

Target		Unit	Target low	Target high
*S*pO$_2$		%	91	93
*P*ETCO$_2$		mmHg	30	60
Protective target region	*V*$_\text {T,pbw}$	ml/kg	4	6
	$\Delta P$	mbar	5	14
Protective acceptable region	*V*$_\text {T,pbw}$	ml/kg	6	8
	$\Delta P$	mbar	5	14
Protective compromise region	*V*$_\text {T,pbw}$	ml/kg	4	6
	$\Delta P$	mbar	14	20

**Fig. 13:**
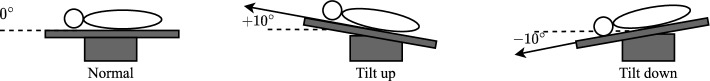
Positional changes are used to provoke disturbances to the system

To test the robustness of the SOLVe system, two kinds of disturbances were introduced during the experiment. First, a brief disconnection (15 s) from the ventilator, simulating routine mucus clearance or an accidental disconnection, causes a rapid de-saturation and lung collapse. Second, the subjects were tilted away from the horizontal position (head-up tilting of $10^{\circ }$ and a head-down tilting of $-10^{\circ }$). This manoeuvre provokes complex changes to blood pressure, ventilation-to-perfusion matching within the lung and large changes in respiratory compliance. A graphical representation is shown in Fig. 13.

## Data Availability

The data sets used and analysed during the current study are available from the corresponding author on reasonable request.

## Abbreviations

ARDS: Acute respiratory distress syndrome
bpm: Breaths per minute
$C_\text {rs}$: Respiratory system compliance
$C_\text {rs,pbw}$: Respiratory system compliance scaled to predicted body weight
CDSS: Computerised decision support system
*F*IO$_2$: Fraction inspired oxygen
$$f_\text {R}$$: Respiratory rate
PCLC: Physiological closed-loop control
PEEP: Positive end-expiratory pressure
*P*ETCO$_2$: End-tidal partial pressure of carbon dioxide
pbw: Predicted body weight
PRVC: Pressure regulated volume control
$R_\text {rs}$: Respiratory system resistance
*S*pO$_2$: Oxygen saturation measured using pulse oximetry
SOLVe: **S**ystem for aut**o**matic **L**ung-protective **Ve**ntilation
VILI: Ventilator-induced lung injury
*V*$$_\text {T}$$: Tidal volume
*V*$_\text {T,pbw}$: Tidal volume scaled to predicted body weight
$$\Delta P$$: Driving pressure
